# Human induced pluripotent stem cell models for Alzheimer’s disease research: a bibliometric analysis

**DOI:** 10.3389/fnhum.2025.1548701

**Published:** 2025-03-19

**Authors:** Yuning Sun, Zhilong Liu, Zongbo Zhang, Yufeng Kang, Xinlian Wang, Yiping Zhang, Yan Liu, Pei Zhao

**Affiliations:** ^1^School of Pharmacy, Gansu University of Chinese Medicine, Lanzhou, China; ^2^Gansu Provincial People’s Hospital, Lanzhou, China; ^3^School of Pharmacy, Lanzhou University, Lanzhou, China

**Keywords:** Alzheimer’s disease (AD), human induced pluripotent stem cell (hiPSC), bibliometrics, inflammation, apolipoprotein E (ApoE)

## Abstract

**Introduction:**

Alzheimer’s disease (AD), the leading cause of dementia, remains without adequate treatment. Current models do not fully replicate human physiology and pathology. The advent of human induced pluripotent stem cell (hiPSC) technology offers a novel approach to studying AD.

**Methods:**

Our study conducted a bibliometric analysis to assess the application and development of hiPSC technology in AD research. We retrieved 531 articles on hiPSC models of AD from the Web of Science Core Collection, published between January 2010 and June 2024. CiteSpace and VOSviewer were used to analyze authorship, geographic contributions, journal influence, and citation patterns.

**Results:**

Our findings reveal a steady increase in publications over 14 years, with the United States leading in contributions, followed by China. Li-Huei Tsai from the Massachusetts Institute of Technology is a prominent researcher. *PLoS One* emerges as the most influential journal. Research trends have focused on inflammation, astrocytes, microglia, apolipoprotein E (ApoE), and tau.

**Discussion:**

Bibliometric analysis is crucial in identifying research gaps and trends and guiding future studies to address unmet needs in understanding and modeling human physiology and pathology. Leveraging hiPSC models to investigate the molecular mechanisms of familial and sporadic AD is expected to provide a crucial foundation for developing future treatment strategies.

**Conclusion:**

In summary, the bibliometric findings from this study provide a comprehensive overview of the current research landscape in hiPSC models for AD. It also highlights emerging trends and research gaps, crucial for guiding future research efforts, particularly in exploring novel therapeutic targets and improving understanding of disease mechanisms.

## 1 Introduction

Alzheimer’s disease (AD) is a prevalent neurodegenerative disorder characterized by progressive cognitive decline and dementia (Yang and Wang, 2022). The pathogenesis of AD remains unclear but is believed to be associated with multiple factors, including genetics, environment, and lifestyle. Current mainstream theories suggest that amyloid-beta (Aβ) deposition, tau protein and neurofibrillary tangle accumulation, oxidative stress, and metal ion dysregulation play significant roles in the disease’s development (Tran et al., 2022). With an aging population, the incidence of AD is rising globally, making it an important public health concern. An estimated 6.9 million Americans aged 65 and older currently have AD, a number projected to grow to 13.8 million by 2060. This disease not only severely impacts patients’ daily lives but also imposes a substantial economic burden on families and society, with total payments for dementia care expected to reach about $360 billion in 2024 (Haines, 2021; Alzheimer’s Association Report, 2024). Current treatments aim to slow progression and improve patients’ quality of life, highlighting the importance of early diagnosis and research into pathogenesis (Jung et al., 2021).

The models currently used in AD research include cell models, brain organoid models, *Caenorhabditis elegans* models, fruit fly models, zebrafish models, and rodent models, with some research employing non-human primate models.

Rodent models have traditionally been extensively used in studying AD mechanisms and have provided essential insights into its pathogenesis (Ifediora et al., 2024). However, while these models capture the complexity of the internal environment, they lack particular disease features specific to humans—the heterogeneity of human diseases and have shorter lifespans, which may not be sufficient to develop neurodegeneration or other critical pathological changes (Karran and De Strooper, 2016; Yang et al., 2021; Tu et al., 2023). The heterogeneity of human diseases—driven by genetic diversity, e.g., apolipoprotein E (ApoE) isoforms, epigenetic modifications, and environmental factors—necessitates models that recapitulate patient-specific pathologies. Human induced pluripotent stem cells (HiPSCs) address this challenge by enabling the generation of patient-derived neurons, glia, and organoids that retain donor-specific genetic and molecular signatures. This allows for the generation of personalized disease models that recapitulate individual patient phenotypes, thereby enabling researchers to dissect the mechanistic diversity underlying AD and pave the way for personalized therapeutic strategies.

HiPSCs have many applications in basic research, drug development, and regenerative medicine. In recent years, hiPSCs models have been increasingly used in AD research, offering a novel approach to establishing human models that reflect human genetics and physiology. This model also provides new insights into the pathogenesis of AD and facilitates the development of candidate drugs for its treatment (Tcw, 2019; Wang et al., 2022). Despite the widespread use of hiPSCs models in AD research, a systematic review has yet to evaluate the relevant literature’s research characteristics and development trends. Bibliometrics is an applied science that emerged in the 1960s (Groos and Pritchard, 1969). During this period, the information explosion and the advancement of computers provided technical support for the analysis of scientific communication. Responding to the need for efficient scientific communication, researchers utilized statistical methods to quantitatively analyze the scientific literature’s development trends (Mayr and Scharnhorst, 2014). Bibliometric studies employ statistical methods for quantitative analysis of scientific literature, which can identify underexplored areas and highlight emerging priorities by analyzing publication trends, citation networks, and keyword co-occurrence. This approach informs funding agencies and policymakers and guides researchers toward high-impact, translational studies addressing critical AD understanding and treatment gaps. It offers a powerful tool to map the evolution of hiPSCs models in AD research. It helps us understand the dynamics of specific fields through visual means and plays a crucial role in predicting future directions (Zhiguo et al., 2023). Consequently, analyzing the trends and application status of research articles in this field through bibliometrics is vital.

In this study, we searched the Social Sciences Citation Index (SSCI) and Science Citation Index Expanded (SCIE) from the Web of Science (WOS) database. WOS, an authoritative and extensive digital literature database, is widely recognized by researchers for its high-quality content. It is considered the most suitable database for bibliometric analysis, essential for evaluating academic achievements and influence. We analyzed the literature on hiPSCs models in AD research published from 2010 to 2024. Based on this literature analysis, we reviewed research hotspots and trends using hiPSCs models for AD and proposed future research directions.

## 2 Methods

### 2.1 Bibliometrics and its tools

Bibliometrics applies mathematical and statistical methods to analyze scientific literature, helping researchers uncover trends and patterns in scientific development. With the growing competition in academic research, bibliometrics has also become significant for educational evaluation. It allows researchers to understand and interpret data from scientific literature more comprehensively through two key approaches: performance analysis and visual analysis. Performance analysis evaluates the validity and reliability of bibliometric indicators, such as citations, the H-index, and impact factors, ensuring these methods accurately reflect the quality and impact of research outcomes (Abramo et al., 2011). The visual analysis uses graphics and visual elements to present bibliometric data, revealing trends and associations through visualization, which helps researchers intuitively understand complex scientific literature data. As information technology advances, bibliometric methods and tools evolve, providing more diverse perspectives and approaches for scientific research and academic evaluation.

CiteSpace and VOSviewer are widely used tools for mapping and analyzing knowledge networks and research trends in scientific literature. CiteSpace excels at analyzing citation networks, revealing relationships among scientific literature. Its time-series view clearly illustrates the development and evolution of research fields, and its algorithms simplify complex network data, making it easier to understand (Chen, 2006). VOSviewer provides highly intuitive visualizations, assisting users in identifying and analyzing significant clusters and associations within a research field, especially in network analysis and clustering (van Eck and Waltman, 2009). These two tools have distinct advantages in data import, processing, and analysis, and when used together, they complement each other to provide more comprehensive analysis results.

### 2.2 Data sources and data collection

The Web of Science Core Collection was selected as the data source in this study. The SCI-Expanded and Social Science Citation Index (SSCI) was chosen to ensure the retrieved data’s comprehensiveness and accuracy. Articles were selected through a three-step process: (1) keyword search in WOS (2010–2024), (2) exclude Review Article, the Editorial Material, and the Abstract, Book Chapters, Correction, Proceeding Paper, News Item, and (3) title/abstract screening to exclude Non-English papers, Redundant publications, Titles/abstracts were focused on non-AD neurodegenerative diseases (e.g., Parkinson’s disease), Lacked explicit use of hiPSCs, non-research articles (e.g., editorials, conference abstracts). The search strategy used in this study is detailed in Supplementary Table S1.

## 3 Results

### 3.1 Basic quantitative information

**Figure 1:**
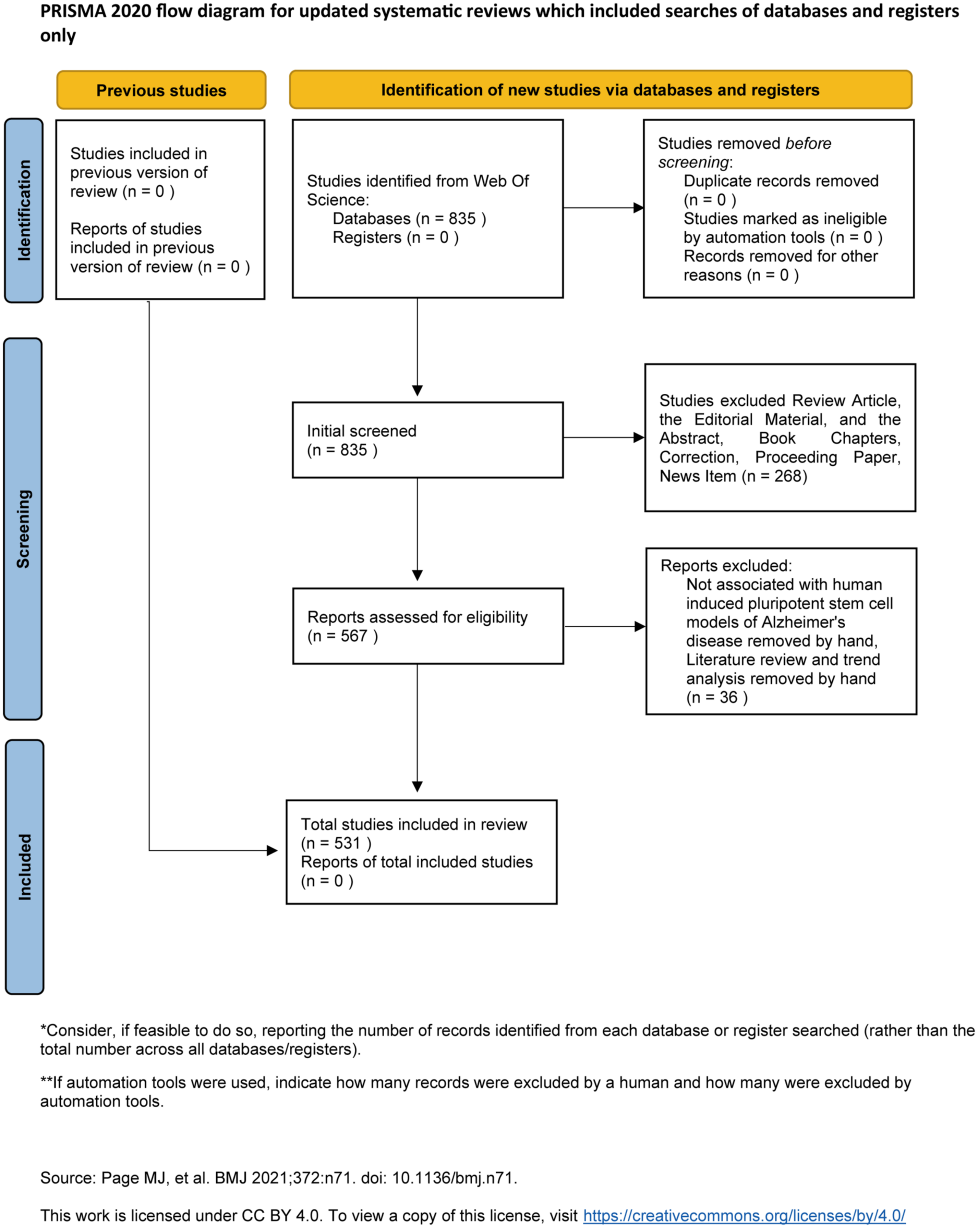
The article selection process for bibliometric mapping analysis.

After de-duplicating the search results, 567 journal papers were identified. After the screening, 531 valid papers were included in the final analysis (Figure 1). The 531 papers analyzed in this study were authored by 3,999 researchers from 957 organizations across 53 countries. These papers were published in 171 journals and received 20,999 citations from 2,314 journals.

### 3.2 Publication volume information

**Figure 2:**
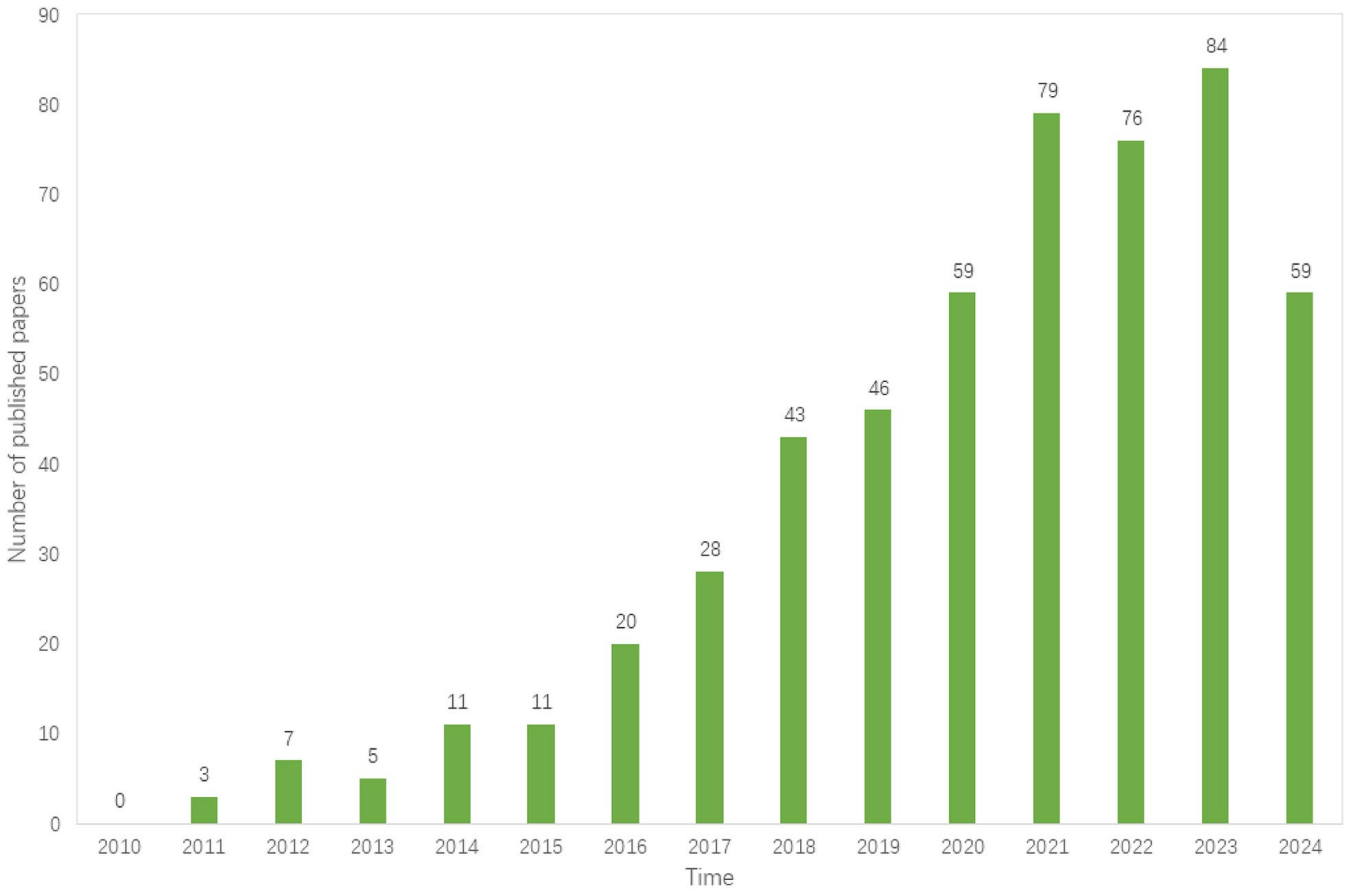
The trend of the publications from 2010 to 2024.

The distribution of publication dates for papers on the hiPSCs model in AD research is from 2010 to 2024 (Figure 2). Overall, the number of documents in this field has increased, with a significant rise observed after 2015. In 2021, 2022, and 2023, the number of published papers stabilized at around 80 per year, indicating that the hiPSCs model in AD research has garnered increasing attention from scholars and has become a focal point in the field.

### 3.3 Author/journal/country

#### 3.3.1 Bibliometric analysis of the author

In 2006, Japanese scientist Shinya Yamanaka discovered induced pluripotent stem cells (iPSCs) by successfully reprogramming mouse fibroblasts into pluripotent stem cells (Takahashi and Yamanaka, 2006). The following year, Yamanaka’s and American scientist James Thomson’s team independently reprogrammed human adult cells into iPSCs, marking a breakthrough in stem cell research and application (Mai and Ellenbogen, 2008). HiPSCs, which utilize genetic engineering techniques to reprogram adult cells into a pluripotent state resembling embryonic stem cells (ESCs), offer a significant advantage: they can be cultured to provide an ample supply of cells without the ethical and practical concerns associated with ESCs derived from early-stage embryos (Lewandowski and Kurpisz, 2016). HiPSCs can be differentiated into neurons to study abnormalities in Alzheimer’s patients and simulate individualized pathological changes (Pen and Jensen, 2017). This advancement enhances our understanding of AD subtypes, enables in-depth characterization of nerve cells in patients with familial and sporadic AD, and more accurately represents the human genome, aiding personalized treatments based on specific genetic backgrounds and disease characteristics (Sally and Cowley, 2016; Beghini et al., 2024; Mungenast et al., 2016; Albert et al., 2021).

**Figure 3:**
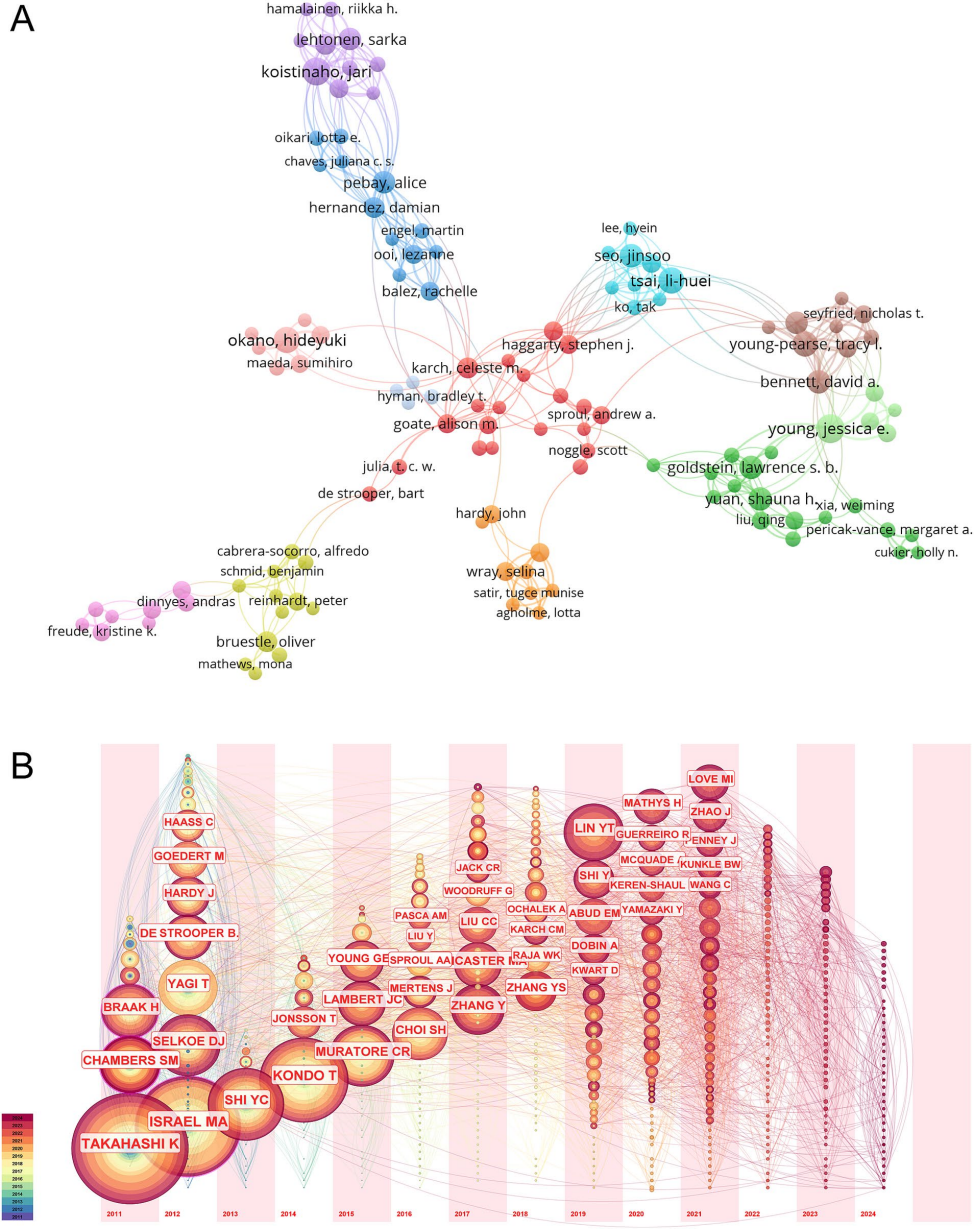
Bibliometric analysis of the authors. **(A)** Co-occurrence network of authors in the field of hiPSCs model research for AD. The network illustrates collaborative relationships among authors, with node size representing the number of publications and line thickness indicating the frequency of collaboration. The cluster sizes represent the number of authors who frequently collaborate. Larger cluster sizes indicate more extensive collaborative networks, potentially highlighting influential research groups. The co-authorship clusters highlight key research hubs and cooperative networks, reflecting shared research interests and potential interdisciplinary collaborations. **(B)** The timezone view of co-citation authors.

Analyzing the authors of the articles provides insights into the key scholars and core research groups in the field of hiPSCs model research for AD. Among the authors listed in Supplementary Table S2, those with over 10 publications are highlighted. Jessica E. Young from the University of Washington stands out, with 13 papers published between January 2010 and June 2024. Her work has been cited 535 times, averaging 41 citations per article. Li-Huei Tsai from the Massachusetts Institute of Technology has an impressive citation record, with 1,672 citations across her published articles, averaging 152 citations per article. Using VOSviewer, an author collaboration network was generated, revealing that 242 authors who published more than three articles formed 12 clusters. The top three authors by total link strength (the number of co-occurrences with other authors) were Jari Koistinaho (the University of Eastern Finland), Alice Pebay (the Melbourne Medical School), and Tracy L. Young-Pearse (Harvard Medical School) (Figure 3A).

The timezone view of co-cited authors, as depicted in Figure 3B, was generated using CiteSpace software. Nodes represent cited authors, and the Slice Length is set to 1, with the Selection Criteria set to the top 50 per slice. Each period corresponds to a vertical time axis, where the author’s name indicates their first appearance in that period. The node size represents the citation frequency, and the lines indicate co-occurrences. Notably, the most frequently cited author is Japanese scholar Kazutoshi Takahashi, whose contributions to studying hiPSCs models for AD are substantial. Takahashi’s 2013 article, “Modeling AD with iPSCs reveals stress phenotypes associated with intracellular Aβ and differential drug responsiveness,” has been cited 327 times (Kondo et al., 2013). A detailed analysis of citation trends reveals that Takahashi’s work gained significant attention in 2017, 2020, 2021, and 2023. The next most frequently cited author is Mason A. Israel, who has also made significant contributions to this field. His article, “Probing sporadic and familial AD using induced pluripotent stem cells,” has been cited 549 times (Israel et al., 2012). Israel’s research saw increased attention between 2018 and 2020. Additionally, Yingsha Zhang received considerable attention in 2024, and Yuan-Ta Lin’s research was widely cited from 2019 to 2023, making notable contributions to the study of hiPSCs models for AD.

#### 3.3.2 Bibliometric analysis of the journal

An analysis of the journals in which these articles were published reveals that most of the research on hiPSCs models for AD over the past decade has been in cell and molecular biology. The top 11 journals ranked by the number of articles published are detailed in Supplementary Table S3. The top three journals are *Stem Cell Research*, *Stem Cell Reports*, and *Scientific Reports*, with 67, 18, and 18 papers, respectively. All three are open-access journals, significantly promoting research progress in this field. Citation analysis shows that *PLoS One* and *Stem Cell Reports* are among the top journals, with 94 and 56 citations, respectively, indicating the high quality and impact of the articles published in these journals.

Bradford’s law describes the distribution pattern of literature in journals on a specific topic. The core region contains a few journals with many articles, the relevant region includes a moderate number of journals and articles, and the peripheral region contains many journals with a few articles. When the number of papers in each area is equal, the ratio of core journals to successive regions is 1:*a*:*a*^2^ (Brookes, 1985). Supplementary Table S4 shows the distribution of documents in the field of hiPSCs model research for AD from 2010 to 2024, where the ratio of the number of journals in the three subdivisions is approximately 1:3.8:3.82, aligning with Bradford’s law.

#### 3.3.3 Bibliometric analysis of countries

**Figure 4:**
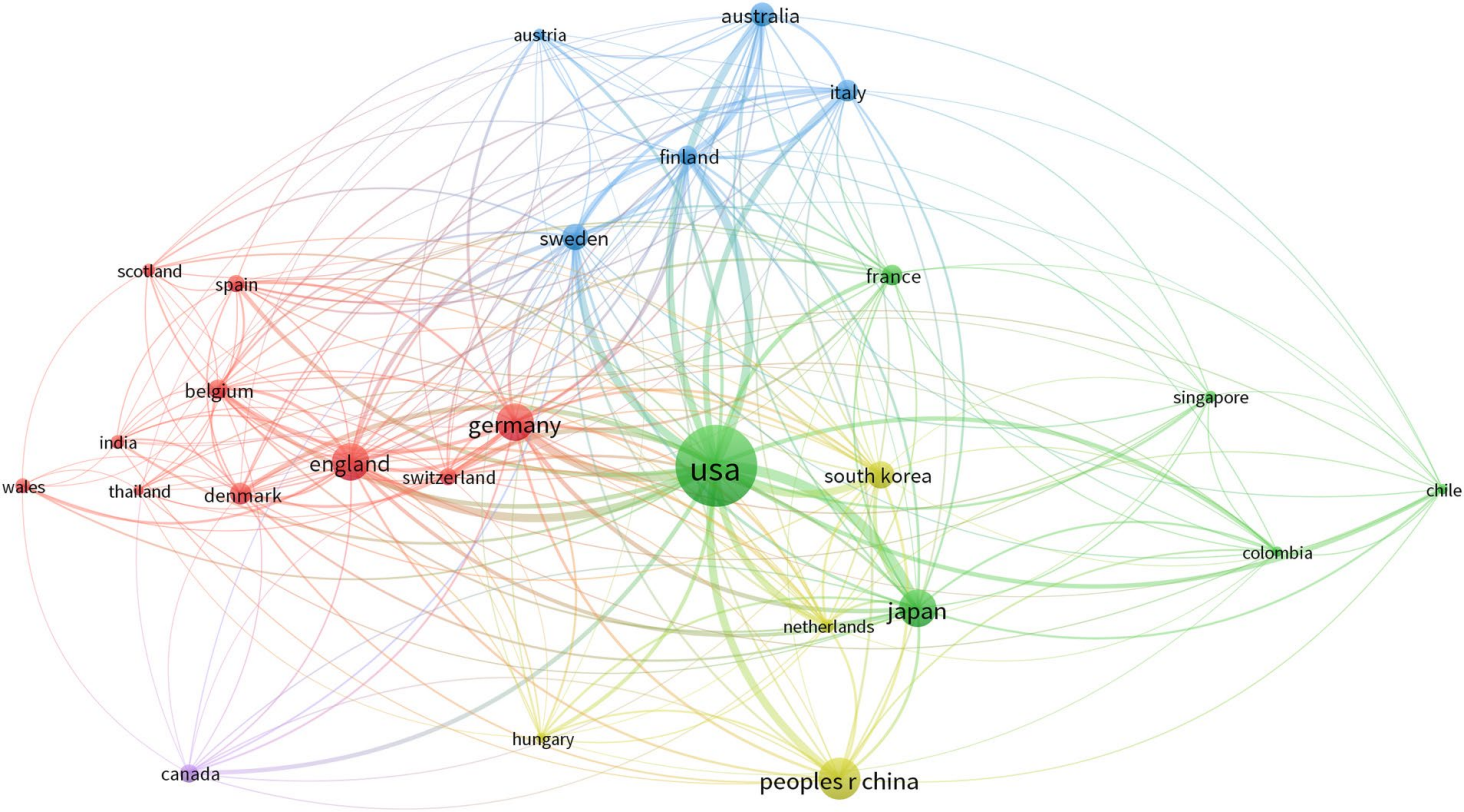
Bibliometric analysis of the nations. Co-occurrence network of countries in the research field of hiPSCs model research for AD. A larger cluster size indicates a broader collaborative network. Thicker lines indicate more frequent cooperation.

To identify which country has made the most significant contribution to the field of hiPSCs model research for AD, the number of publications from 53 countries was analyzed. The top 10 countries are listed in Supplementary Table S5. The United States leads with 273 articles, accounting for 51.4% of the total, with an average of 51 citations per article. China follows with 80 articles. Denmark has the highest number of citations per article, averaging 68. Denmark exhibits the highest citation rate per article, surpassing countries with higher publication volumes. This exceptional impact stems from Denmark’s focus on high-risk/high-reward research (e.g., ApoE-driven lipid dysregulation), strategic international collaborations, and specialization in emerging subfields like neuroinflammation. High-productivity countries with five or more published papers were analyzed and visualized using VOSviewer. The results indicate an uneven distribution of publishing countries, with a significant concentration of contributions from a few top countries, such as the USA, China, Japan, and Germany (Figure 4).

### 3.4 Analysis of keywords

#### 3.4.1 Keyword co-occurrence network analysis

**Figure 5:**
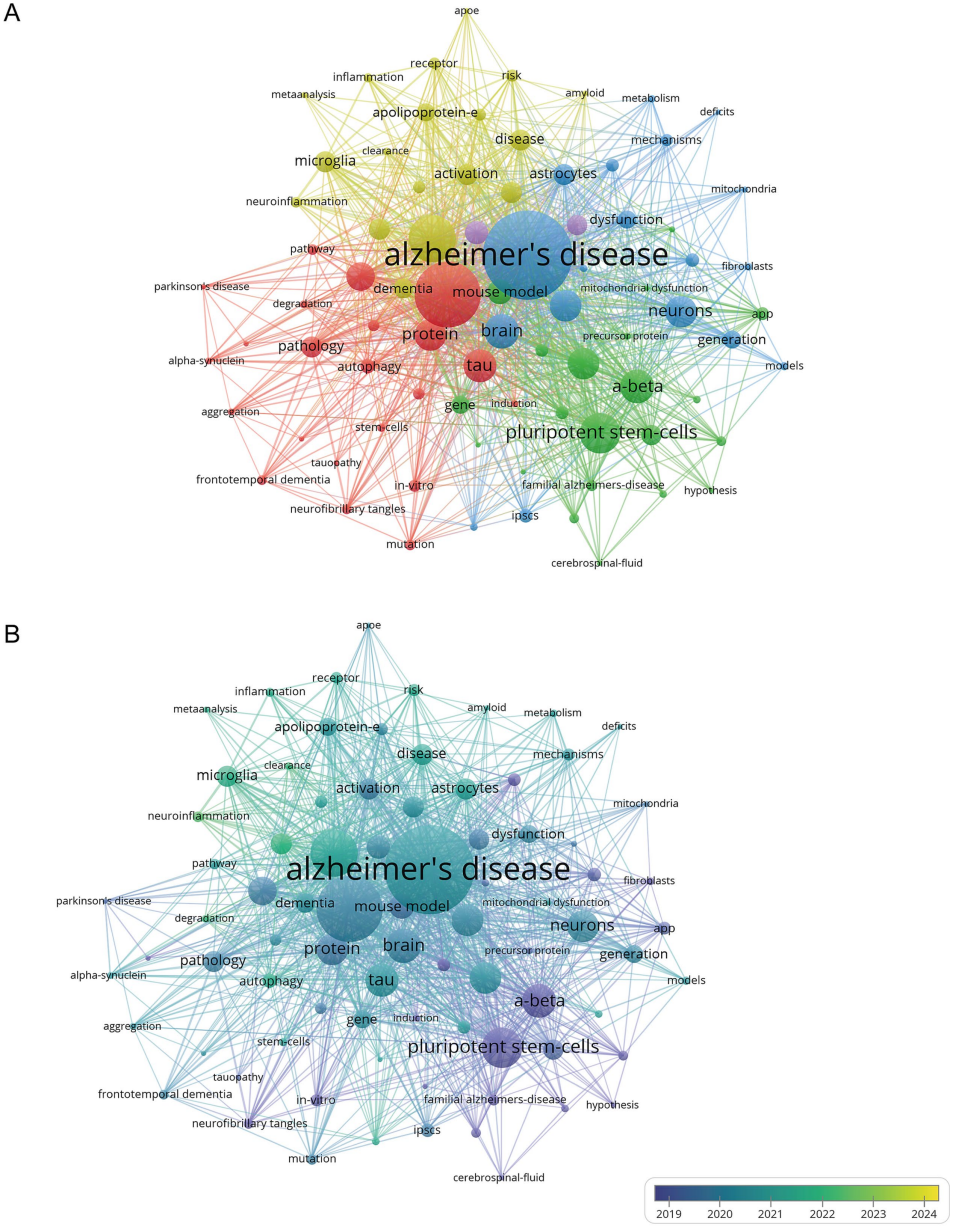
Bibliometric analysis of the keywords. **(A)** Cluster network of keywords in the research field of hiPSCs model research for AD. **(B)** Keyword time view in the research field of hiPSCs model research for AD.

Keyword co-occurrence analysis reveals the relationships between keywords and the knowledge structure within a field by analyzing the frequency of keyword co-occurrence in papers. This analysis helps identify research hotspots and guide future research directions. VOSviewer was used to create a keyword co-occurrence network for 531 publications, selecting 81 keywords with a frequency of 10 or more for visualization. The larger the nodes in the visualization, the more frequently the keywords appear, indicating research hotspots. Keyword clusters are displayed in Figure 5A, with different node colors representing clusters or research topics. The analysis shows that keywords related to the hiPSCs model in AD from 2010 to 2024 are divided into five clusters.

The first cluster (red) centers around secondary core keywords such as protein, tau, neurofibrillary tangles, autophagy, and phosphorylation. The research on this cluster may explore how to slow or stop the pathological progression of AD by regulating tau phosphorylation and/or enhancing autophagy. In AD, tau protein is abnormally hyperphosphorylated, causing it to detach from microtubules and form paired helical filaments (PHFs), eventually accumulating into neurofibrillary tangles. The autophagy pathway may be disrupted, accumulating abnormal proteins such as phosphorylated tau, as the autophagy pathway cannot effectively clear these damaged proteins. This abnormal phosphorylation of tau may affect the autophagy process, and decreased autophagy function further exacerbates the abnormal deposition of tau (Kuang et al., 2019).

The second cluster (green) involves keywords such as amyloid precursor protein (APP), amyloid-beta, familial AD, and presenilin 1. The genetic basis of familial AD (FAD) is closely related to mutations in the APP and PSEN1 genes, which affect APP processing and Aβ production, highlighting the key role of Aβ in the pathogenesis of AD. Presenilin 1 (PSEN1) is easily cleaved by caspase, increasing the amount of Aβ protein in neurons, and its loss-of-function mutation is a significant factor in FAD.

The third cluster (blue) includes keywords like brain, neurons, astrocytes, and oxidative stress. In AD, Aβ can induce oxidative stress in astrocytes, which are involved in the inflammatory and immune responses of the central nervous system (González-Reyes et al., 2017). The fourth cluster (yellow) focuses on keywords such as apolipoprotein E (ApoE), microglia, and neuroinflammation. The activation status of microglia in AD is related to the ApoE genotype, with ApoE4 potentially exacerbating the pro-inflammatory response of microglia and promoting neurodegeneration. ApoE significantly impacts the risk of AD, increasing the risk and lowering the age of onset in a gene-dose-dependent manner, thus severely affecting gene expression in Alzheimer’s patients (Najm et al., 2019; Yamazaki et al., 2019). Microglia play a major role in neuroinflammation, and their activation may affect AD progression (Leng and Edison, 2020; Bairamian et al., 2022).

The fifth cluster (purple) includes keywords related to differentiation and models. Induced pluripotent stem cell technology can reprogram human cells into a pluripotent state and differentiate them into various brain cell types, such as neurons, astrocytes, and microglia. This allows for a more comprehensive analysis of AD complexity by constructing hiPSCs models. Therefore, researchers focused on the different pathogenesis of AD, using the hiPSCs model for AD to study further. High-frequency keywords, those with a frequency greater than 50, are displayed in Supplementary Table S6 to enhance the comprehension of the keywords.

The keyword time view (Figure 5B), where color mapping reflects the average years of keyword usage, highlights research trends. The time frame of 2019–2024 was selected to illustrate keyword trends in papers related to the hiPSCs model of AD. Since 2019, research hotspots have focused on inflammation, neuroinflammation, astrocytes, microglia, autophagy, ApoE, and tau. High-frequency keywords such as amyloid-beta, brain, protein, tau, and neurons emerge as key terms in hiPSCs model research for Alzheimer’s Disease (Figure 5 and Supplementary Table S6). Inflammation, astrocytes, microglia, and ApoE are the main research frontiers in this field.

AD’s most potent genetic risk factor is ApoE, a lipid and cholesterol transporter (Lambert et al., 2013). In recent years, numerous studies have been conducted on the mechanism of ApoE in delayed AD. Studies have found that homozygous R136S mutation can save ApoE4-driven tau pathology, neurodegeneration, and neuroinflammation through tau hiPSC-derived neuron models of human ApoE4 with homozygous or heterozygous R136S mutation. The hybrid R136S mutation has a partial protective effect against ApoE4-driven neurodegeneration and neuroinflammation, which is a massive advance for the treatment of late-onset AD (Nelson et al., 2023). Many studies have emphasized the critical role of ApoE in the mechanism of delayed AD by using human ApoE gene iPSCs, suggesting that we can treat delayed AD by regulating the expression of ApoE.

Recent hiPSC studies have significantly advanced our understanding of tau pathology in AD. For instance, Parra Bravo et al. (2024) established a robust 4R tauopathy model using CRISPR-edited iPSC-derived neurons. They identified critical genetic modifiers of tau propagation through the CRISPR interference (CRISPRi) screening. Their findings on the role of UFMylation and retromer complex components (e.g., VPS29) in tau aggregation and spread provide essential insights into pathways that may influence familial and sporadic AD pathogenesis. Such models are critical for dissecting the spatiotemporal progression of tau pathology and testing anti-tau therapies in human-relevant systems. The rise of “tau” underscores efforts to develop tau aggregation inhibitors. Recent strategies have prioritized tau-centric approaches, including microtubule stabilizers (e.g., davunetide) and anti-phospho-tau immunotherapies (e.g., semorinemab) (Morimoto et al., 2014; Teng et al., 2022).

The growing emphasis on neuroinflammation and glial cells in AD research underscores their pivotal roles in disease pathogenesis and potential therapeutic interventions. Microglia, the brain’s resident immune cells, are central to AD progression. While their primary role involves Aβ clearance and synaptic maintenance, chronic activation in AD leads to a pro-inflammatory phenotype characterized by excessive cytokine release (e.g., IL-1β, TNF-α) and ROS production, exacerbating neuronal damage. Recent hiPSC-derived microglia models have revealed that genetic risk factors such as ApoE4 and Triggering receptor expressed on myeloid cells 2 (TREM2) mutations impair phagocytic function while amplifying inflammatory responses, accelerating Aβ accumulation and tau propagation (Victor et al., 2022; Penney et al., 2023). These findings suggest modulating microglial activity by enhancing Aβ clearance (e.g., via TREM2 agonism) or suppressing maladaptive inflammation (e.g., through NOD-like receptor thermal protein domain associated protein 3—NLRP3 inflammasome inhibition) could mitigate AD progression. Astrocytes, traditionally viewed as metabolic supporters of neurons, are now recognized as dynamic participants in AD pathology. HiPSC-derived astrocytes carrying ApoE4 exhibit disrupted lipid metabolism, impaired Aβ degradation, and heightened secretion of pro-inflammatory mediators, which collectively promote synaptic loss and neurotoxicity (Sienski et al., 2021; Lin et al., 2018). Notably, ApoE4 astrocytes fail to maintain cholesterol homeostasis, leading to myelin dysfunction and neuronal hyperexcitability, a phenotype reversible by gene editing to ApoE3 (Tsai et al., 2023). These observations position astrocyte-targeted therapies, such as ApoE isoform modulation, lipid metabolism restoration, or anti-inflammatory cytokine delivery, as promising avenues for AD treatment. The advent of hiPSC technology has enabled the generation of patient-specific glial cells, offering unprecedented opportunities to dissect the interplay between genetic variants, neuroinflammation, and AD phenotypes. Single-cell transcriptomic analyses of hiPSC models have identified subtype-specific glial responses (e.g., disease-associated microglia, reactive astrocytes), highlighting the need for precision therapeutics tailored to distinct inflammatory states (Keren-Shaul et al., 2017). Integrating hiPSC models into AD research has deepened our understanding of glial contributions to neuroinflammation, unveiling actionable targets for therapeutic development.

The temporal evolution of keyword prominence, particularly the marked increase in “tau” (2018–2024, Figure 5B), signifies a pivotal paradigm shift in AD research—from amyloid-beta (Aβ)-centric frameworks toward tauopathy-driven mechanistic exploration. Accumulating evidence that tau pathology exhibits a stronger spatiotemporal correlation with neurodegeneration and cognitive impairment than Aβ plaques (Ayyubova, 2023). Similarly, the surge in “inflammation”-associated research (2019–2024) mirrors the growing recognition of neuroimmune dysregulation as a critical driver of AD progression.

#### 3.4.2 Evolution analysis on keywords

**Figure 6:**
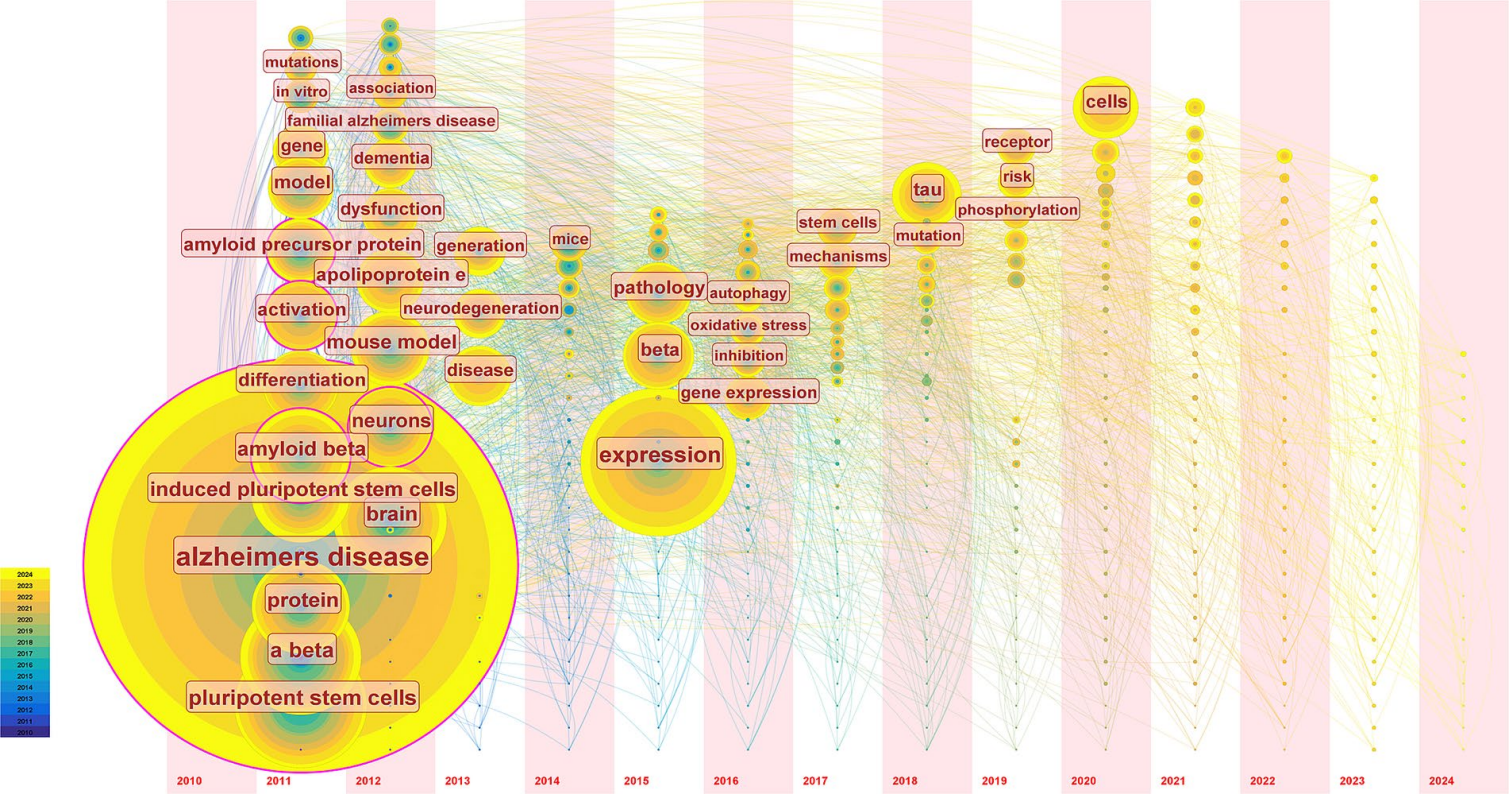
The timezone view of keywords. We extracted data keywords ranked in the top 50 based on frequency and relevance from relevant publications in each time slice and visualized the temporal distribution. Each circle in the graph represents a keyword for the year in which the keyword first appeared in the analyzed dataset. If the keyword appears again in a later year, it will be increased by 1 in the location where it first appeared. The line is the co-occurrence relationship between keywords. The temporal trends reveal a shift in research focus over time, highlighting areas of growing interest and potential future research directions.

Keyword co-occurrence time zone map can be organized chronologically to illustrate research trends and identify the inflection points and temporal patterns in developing hiPSCs models for AD. In this study, we extracted data ranked in the top 50 in each time slice to generate a keyword co-occurrence time zone map (Figure 6). Combined with qualitative analysis, Figure 6 illustrates that research topics on induced pluripotent stem cell models for AD can be divided into three phases: the first phase (2011–2012) focused primarily on Aβ research, involving keywords like amyloid, mutations, familial AD, and ApoE. The second phase (2013–2017) was characterized by more scattered research topics, with low keyword occurrence frequencies, focusing mainly on autophagy and oxidative stress. The third phase (2018-present) shows a continuing divergence in research themes, with the emergence of high-frequency keywords such as tau and phosphorylation analysis of burst words.

To gain a more precise understanding of the bursting keywords in the field of hiPSCs research for AD, CiteSpace’s burst keyword analysis feature was used, combining data from 2010 to 2024 to detect significant keyword bursts. Burst keywords frequently appear within a specific period, revealing the evolution of research hotspots over time and recent trends and potential future directions. The 12 bursting keywords detected are shown in Supplementary Figure S1. These bursts primarily occurred between 2019 and 2022. Mitochondrial dysfunction received increased attention in 2019, indicating that researchers have begun focusing on its role in AD pathology.

### 3.5 Co-citation analysis

**Figure 7:**
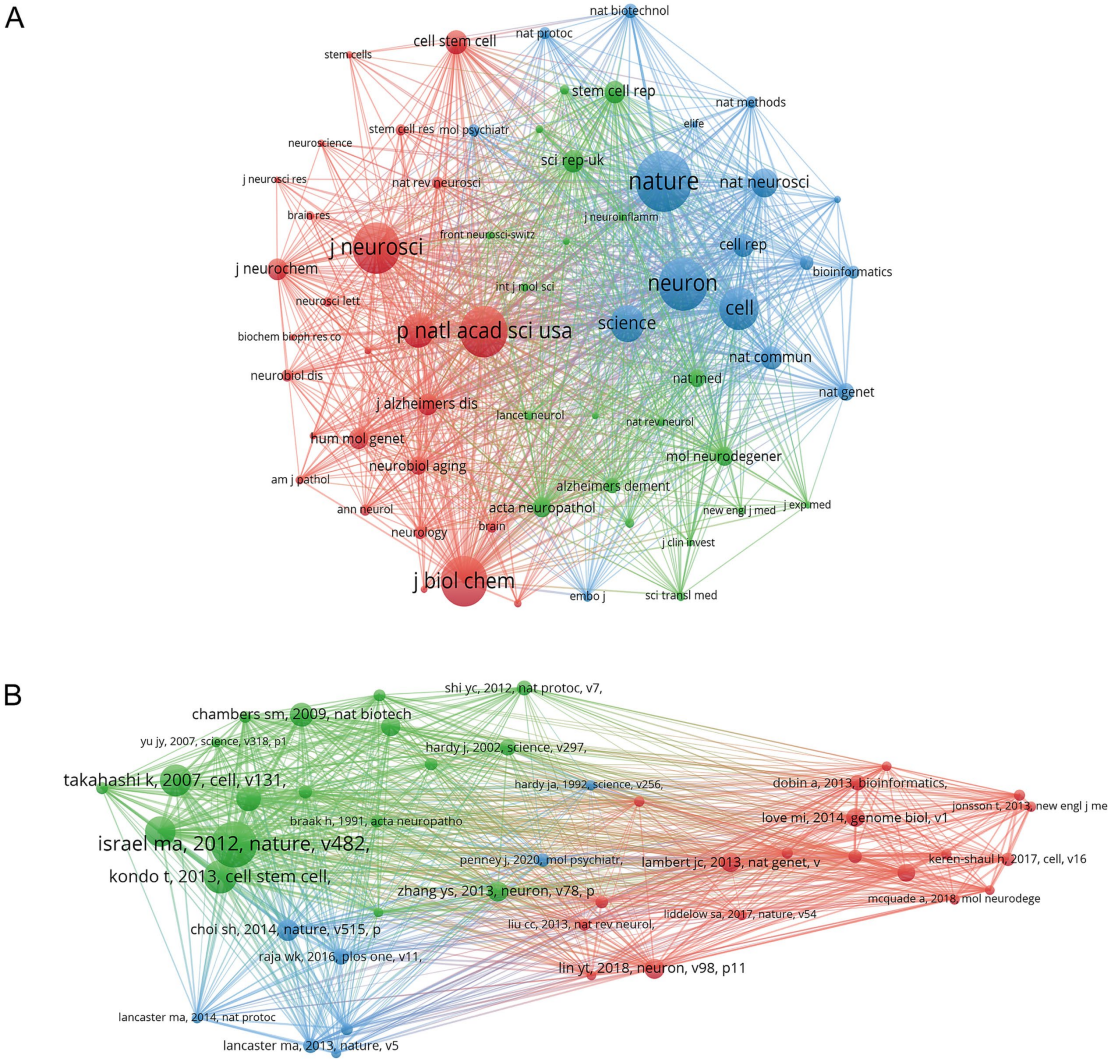
Bibliometric analysis of journals and references. **(A)** Co-citation map of cited journals. **(B)** Co-citation map of cited references.

The purpose of co-citation analysis is to identify the most frequently cited papers and the journals that publish them within the research field of hiPSCs models for AD. Using VOSviewer, we generated a journal co-citation map, setting the minimum threshold for co-citations at 100. This analysis included 63 journals, and the resulting co-citation network is depicted in Figure 7A. The total citation network can be divided into three clusters. The top five journals by citation count are *Nature* (1,003 citations), *Neuron* (863 citations), *Neuroscience* (843 citations), *Proceedings of the National Academy of Sciences of the United States of America* (840 citations), and *Journal of Biological Chemistry* (824 citations). These five journals are all recognized for their high quality.

We further analyzed the co-citations of individual papers. First, VOSviewer was used to identify the top five most-cited papers in this field from 2010 to 2024, as shown in Table 1. Table 1 summarizes the top-cited publications in hiPSC-based AD research, each contributing uniquely to the field. For instance, Israel et al. established hiPSCs as a transformative tool for modeling familial and sporadic AD. At the same time, Kondo et al. leveraged this platform to uncover Aβ-associated stress mechanisms (Israel et al., Probing sporadic and familial AD using induced pluripotent stem cells. Kondo et al. Modeling AD with iPSCs reveals stress phenotypes associated with intracellular Aβ and differential drug responsiveness). These studies are highly cited due to their methodological rigor, mechanistic insights, and translational implications, collectively shaping modern AD research paradigms. Then, we created a reference co-citation graph, setting the minimum co-citation threshold at 20, which resulted in 45 references being included in the analysis (Figure 7B). The co-citation network of highly co-cited papers, as depicted in Figure 7B, is segmented into three distinct clusters. Most of the highly-cited papers were published between 2013 and 2018, with only three papers published after 2019 being mentioned more than 20 times, suggesting that the quality of recent articles in this research field needs improvement.

**Table 1:** Top five publications in the field of hiPSCs models of AD.

Rank	Title	Journal	Year	Citations
1	Probing sporadic and familial AD using induced pluripotent stem cells	*Nature*	2012	93
2	Modeling AD with iPSCs reveals stress phenotypes associated with intracellular Aβ and differential drug responsiveness	*Cell Stem Cell*	2013	68
3	Induction of pluripotent stem cells from adult human fibroblasts by defined factors	*Cell*	2007	65
4	Modeling familial AD with induced pluripotent stem cells	*Human Molecular Genetics*	2011	61
5	The familial AD APPV717I mutation alters APP processing and tau expression in iPSC-derived neurons	*Human Molecular Genetics*	2014	50

## 4 Discussion

This study utilized VOSviewer and CiteSpace software to analyze relevant research on hiPSC models in AD over the past decade, systematically reviewing the development trends in this field. Most of the AD models summarized are presented in the form of reviews, so the bibliometric analysis proposed in this paper is of great value. As research into the mechanisms of AD advances, the importance of hiPSCs in this area has become increasingly emphasized. The steady increase in publications and citations in recent years reflects the growing interest among researchers.

Regarding global contributions, the United States and China lead the way. According to the 2024 Alzheimer’s Facts and Figures report, 6.9 million people age 65 and older have AD, which is now the fifth leading cause of death in the United States. This statistic underscores the critical importance of research in this area (Alzheimer’s Association Report, 2024). Among the most prolific authors, Jessica E. Young from the University of Washington has published the most papers, publishing 13 papers between January 2010 and June 2024, making an undeniable contribution to this field. Li-Huei Tsai of the Massachusetts Institute of Technology stands out as the most influential researcher, with significant contributions to the study of neurodegenerative diseases, particularly AD. Since 2014, Tsai has concentrated on hiPSCs models, publishing numerous high-quality articles in top-tier journals.

Initially, Tsai’s research included using brain organoids derived from hiPSCs from AD patients to study pathological processes such as amyloid aggregation, hyperphosphorylated tau protein, and neurofibrillary tangle formation. Subsequently, Tsai has used hiPSCs to explore the role of p25/Cdk5 in tau pathology, demonstrating that blocking p25 production reduces phosphorylated tau levels and increases synaptophysin expression (Seo et al., 2017). In 2018, Tsai used human ApoE alleles hiPSCs to find that ApoE4 affects AD pathology mainly by damaging astrocytes and microglia-mediated Aβ clearance. In other words, converting ApoE4 into ApoE3 in brain cell types of sporadic AD hiPSCs can attenuate various AD-related pathology, a breakthrough for treating sporadic AD (Lin et al., 2018). Later, Tsai further studied the regulatory role of ApoE4 in lipid transport and metabolism in AD. It was found that the cellular liposomes of hiPSC-derived ApoE4 astrocytes were destroyed. That choline was added to hiPSC-derived astrocytes expressing ApoE4 and yeast expressing human ApoE4 to restore the cellular lipid set to its elemental state (Sienski et al., 2021). Based on previous studies, Tsai used hiPSCs to study the effect of ApoE4 on the bidirectional communication between neurons and microglia. She found that ApoE4-induced lipid accumulation made microglia respond less to neuronal activity (Victor et al., 2022). Tsai also used hiPSCs from SAD to explore the pathogenesis of SAD and found that neural progenitor cells from the SAD iPSC line showed significantly increased expression of genes related to neural differentiation, leading to premature neuronal differentiation and reduced NP cell renewal (Meyer et al., 2019). In 2024, Tsai and colleagues characterized TREM2 R47H/+ mutations that have some potentially deleterious effects on microglia gene expression and function by using gene editing and hiPSC-based microglia models *in vitro* and *in vivo*, promoting inflammation and synaptic loss (Penney et al., 2023). It can be seen from the above that Tsai has made great efforts to use the hiPSCs model to study the related mechanisms of AD and will continue to make substantial contributions to the understanding and potential treatment of AD.

This study found that the research focus on hiPSCs models in AD has recently shifted toward inflammation, neuroinflammation, astrocytes, microglia, ApoE, and tau. The dominance of inflammation, tau, and ApoE underscores a strategic reorientation toward multifactorial AD mechanisms, with significant implications for drug development and precision medicine. Tau-targeted therapeutic strategies, exemplified by semorinemab and davunetide, are progressing through clinical trials. HiPSC models facilitate the screening of tau modifiers and the optimization of drug efficacy, offering critical clinical insights to guide future drug development endeavors (Parra Bravo et al., 2024; Teng et al., 2022). Similarly, the researchers studied the pathological mechanisms associated with TREM2 based on HiPSC-derived glia models. They used these models to optimize anti-inflammatory strategies, such as developing agonists that target the TREM2 receptor (Penney et al., 2023; Long et al., 2024). ApoE4-focused interventions, including gene editing and lipid modulators, exemplify the potential of hiPSC models to guide personalized therapies. While these trends align with the field’s shift from Aβ-centric models, they also highlight critical gaps. For instance, the emphasis on inflammation risks neglecting vascular and mitochondrial pathologies, contributing to AD heterogeneity.

Despite the strong correlation between tau and cognitive decline, which offers potential for therapeutic interventions, the translation of these findings into effective treatments is impeded by significant challenges in drug delivery. The complexity of delivering therapeutic agents to the brain in tauopathies is attributed to the blood-brain barrier, the intracellular localization of tau, and the size and complexity of tau aggregates (Congdon et al., 2023). These factors necessitate the development of innovative delivery mechanisms, optimizing agents for brain penetration and neuronal uptake, and strategies to overcome barriers posed by tau structure and function. Addressing these drug delivery challenges is crucial for successfully translating tau-targeting therapies from the laboratory to the clinic. Conversely, ApoE-focused research exemplifies successful bench-to-bedside translation. Balancing mechanistic depth with therapeutic feasibility will be key to advancing AD research.

HiPSC models offer a promising avenue to tackle the significant challenges inherent in AD research, including disease heterogeneity, the deficiency of personalized therapeutic strategies, and the limited availability of novel therapeutic targets beyond Aβ and tau. HiPSC models hold transformative potential for bridging AD research and clinical translation. First, hiPSCs enable patient-specific disease modeling that recapitulates genetic diversity and sporadic AD heterogeneity. This approach allows clinicians to stratify patients based on *in vitro* phenotypes, such as the application of ApoE4 gene editing therapy, which can be explored as a potential intervention strategy to mitigate these phenotypic defects. Furthermore, hiPSC platforms accelerate drug discovery by prioritizing human-relevant therapeutic targets. Finally, hiPSC-derived neurons provide insights into biomarker discovery, as dysregulated pathways (e.g., oxidative stress, autophagy) correlate with cerebrospinal fluid biomarkers like phosphorylated tau and Aβ_42/40_ ratios. These models may also uncover novel non-invasive biomarkers, such as astrocyte-secreted miRNAs or lipid metabolites. Collectively, hiPSC technology advances precision medicine, drug development, and early diagnosis, positioning it as a cornerstone of translational AD research.

However, hiPSCs models also have limitations, such as the inability to fully replicate the brain’s microenvironment and challenges in creating and maintaining these models under specific experimental conditions. Somatic cell reprogramming into iPSCs resets the epigenetic landscape, which may erase epigenetic and aging markers critical for modeling late-onset neurodegenerative disorders like AD. To mitigate this limitation, emerging approaches such as direct reprogramming (or transdifferentiation) offer a promising alternative. Direct reprogramming converts somatic cells (e.g., fibroblasts) into functional neurons or glial cells by bypassing the pluripotent state. For instance, Gage and colleagues demonstrated that directly reprogrammed neurons retain donor age-associated transcriptional and epigenetic features, enabling the study of aging-related mechanisms in neurodegeneration (Mertens et al., 2015). HiPSC models face significant limitations in replicating the complexity of AD pathology. First, conventional 2D hiPSC-derived neurons and glia lack the three-dimensional (3D) architecture and multicellular interactions (e.g., neuron-microglia-astrocyte crosstalk) critical for modeling AD pathology (Kelley and Pasca, 2022). The brain microenvironment includes vascular networks and resident immune cells (e.g., microglia), absent in traditional hiPSC models. This limits their ability to study neurovascular dysfunction or neuroinflammation driven by peripheral immune infiltration. Emerging 3D organoid technologies address these gaps by enabling the generation of self-organized neural tissues with diverse cell types and layered structures.

For example, human pluripotent stem cell-derived brain organoid techniques can be used to model familial Alzheimer’s disease (Choe et al., 2024). Vascularized organoids co-cultured with endothelial cells and microglia further model blood-brain barrier breakdown and immune cell infiltration. These advancements, coupled with CRISPR-based high-throughput screens, position 3D organoids as a transformative tool for dissecting AD mechanisms and accelerating therapeutic discovery. Second, while familial AD mutations are readily modeled, sporadic AD’s polygenic and environmental heterogeneity remains challenging to recapitulate. Future studies could bridge these gaps by integrating 3D organoid-vascular co-cultures and multi-omics profiling (Gulati et al., 2020).

## 5 Conclusion

In summary, hiPSCs technology has broad applications in AD research, offering great potential for understanding the disease’s pathogenesis and developing early diagnosis and treatment strategies. While Aβ and tau remain central, the underrepresentation of neurovascular and mitochondrial mechanisms underscores the need for holistic models that capture AD’s multifactorial nature. The rising emphasis on neuroinflammation and ApoE4 offer actionable pathways for innovation, such as glia-targeted therapies and genotype-stratified clinical trials. The stratification of clinical trials is essential for accurately assessing the efficacy of new therapeutic interventions. The heterogeneity of AD, driven by genetic factors such as ApoE4 and diverse pathological mechanisms involving tau and neuroinflammation, complicates the design and implementation of clinical trials. Future studies should focus on developing biomarker-based stratification strategies to identify patient subgroups most likely to benefit from specific interventions. Genetic profiling, including ApoE genotyping, can refine trial stratification by identifying individuals with specific genetic risk factors. The development of imaging techniques, such as PET scans targeting tau and amyloid plaques, can provide non-invasive means to monitor disease progression and treatment response in real time. Despite ongoing challenges, particularly in early diagnosis, the continued exploration and innovation by researchers worldwide hold promise for the effective prevention and treatment of AD in the future.

## 6 Limitation

This bibliometric study still has some limitations. First, while we used the Web of Science database, which is widely recognized, other databases might provide additional important information. Second, only English-language literature was included, which may have excluded significant studies published in different languages. The Web of Science Core Collection was selected as the data source in this study due to its high-quality citation data and comprehensive coverage of multidisciplinary research. WOS is particularly well-suited for bibliometric studies due to its rigorous indexing standards and the inclusion of high-impact journals and emerging research areas. For this study, it is considered the most suitable database for bibliometric analysis, essential for evaluating academic achievements and influence. While PubMed is a valuable resource for biomedical research, it primarily focuses on the life sciences and medicine, which may limit its coverage of interdisciplinary research areas.

In contrast, WOS provides more extensive and multidisciplinary coverage, which is particularly advantageous for our study. Furthermore, PubMed does not offer the same depth of citation analysis, making it less suitable for our specific research objectives. Our study focused on English-language publications to ensure consistency in bibliometric analysis, as English is the dominant language in international scientific communication. This approach aligns with standard practices in bibliometric studies aiming to map global research trends. Our reliance on WOS and exclusion of non-English literature may introduce geographic and thematic biases. Non-English studies from non-Anglophone regions may emphasize distinct research priorities, such as traditional medicine or region-specific AD risk factors. For instance, Japanese studies on herbal compounds for neuroprotection or Chinese research on ApoE subtypes in Asian populations might be underrepresented. By excluding these, our analysis may overlook culturally tailored therapeutic approaches. Platforms like CiNii (Japan) or CNKI (China) host studies on hiPSC models optimized for local genetic backgrounds, which are absent in WOS. This could skew our bibliometric trends toward Western-centric research paradigms, highlighting the need for multi-platform analyses in future work. While our methodological choices prioritize consistency and feasibility, we agree that future studies could enhance comprehensiveness by integrating multilingual databases and cross-platform validation, strengthening our work’s transparency and rigor. Lastly, the data analyzed were current up to June 30, 2024. Articles published more recently may still need sufficient citations to represent current trends.

## Data Availability

The original contributions presented in the study are included in the article/supplementary material, further inquiries can be directed to the corresponding authors.
