# Supplementary material for: Human induced pluripotent stem cell models for Alzheimer’s disease research: a bibliometric analysis
Source: Front Hum Neurosci. 2025 Mar 19;19:1548701. doi: 10.3389/fnhum.2025.1548701 (PMC11962003; doi:10.3389/fnhum.2025.1548701)
Supplement: Supplementary file 1 [file Data_Sheet_1.docx]

Supplementary Material

**
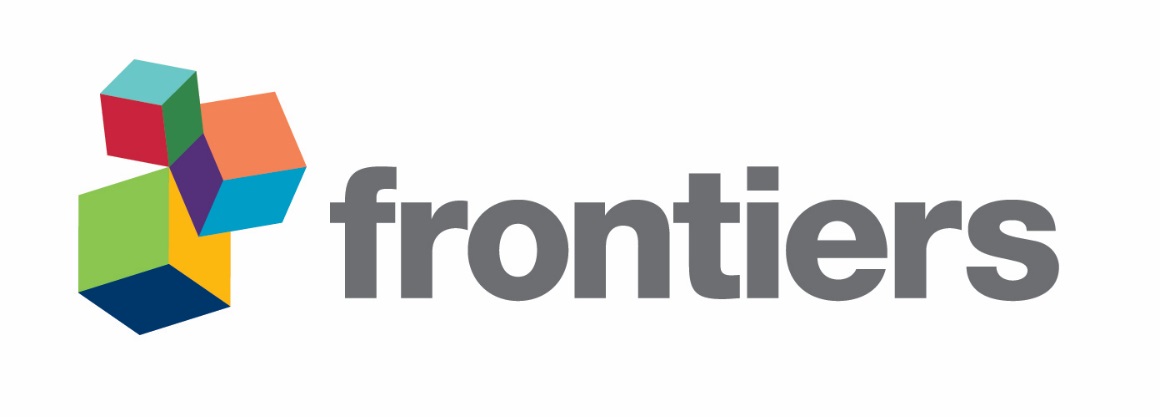
**

**Supplementary Figure 1.** The burst years and intensity of burst words.


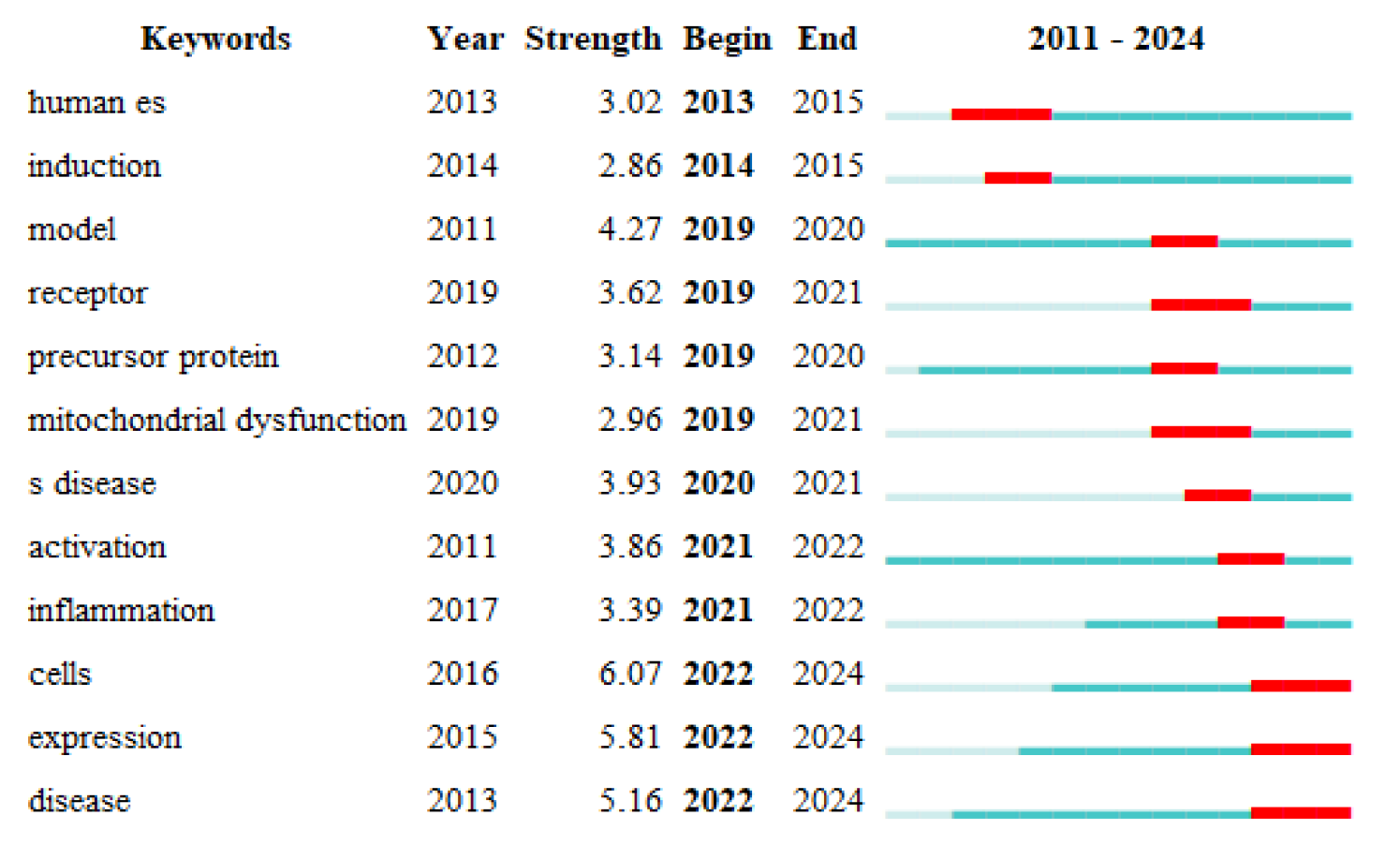


**S1 Table. Summary of data source and selection.**

| Category | Specific standard requirements |
| --- | --- |
| Research database | Web Of Science core collection |
| Citation indexes | SCIE and SSCI |
| Searching period | 2010-01-01 to 2024-06-30 |
| Language | English |
| Searching keywords | TS=（"Alzheimer Disease" OR "Alzheimer Syndrome" OR "Alzheimer-Type Dementia" OR "Alzheimer Type Dementia" OR "Dementia, Alzheimer-Type" OR "ADs" OR "Alzheimer Diseases" OR "Alzheimers Diseases" OR "Alzheimer Dementia" OR "Alzheimer Dementias" OR "Dementia, Alzheimer" OR "AD" OR "Dementia, Senile" OR "Senile Dementia" OR "Dementia, Alzheimer Type" OR "Alzheimer Type Dementia" OR "Senile Dementia, Alzheimer Type" OR "Alzheimer Type Senile Dementia" OR "Primary Senile Degenerative Dementia" OR "Alzheimer Sclerosis" OR "Sclerosis, Alzheimer" OR "Dementia, Primary Senile Degenerative" OR "Dementia, Presenile" OR "Presenile Dementia" OR "Acute Confusional Senile Dementia" OR "Senile Dementia, Acute Confusional" OR "Alzheimer Disease, Early Onset" OR "Early Onset Alzheimer Disease" OR "Presenile Alzheimer Dementia" OR "Alzheimer Disease, Late Onset" OR "Late Onset Alzheimer Disease" OR "AD, Focal Onset" OR "Focal Onset AD" OR "Familial Alzheimer Disease" OR "Alzheimer Disease, Familial" OR "Familial Alzheimer Diseases"）AND（"Induced Pluripotent Stem Cells" OR "IPS Cell" OR "Cell, IPS" OR "Cells, IPS" OR "Induced Pluripotent Stem Cell" OR "IPS Cells" OR "Fibroblast-Derived Induced Pluripotent Stem Cells" OR "Fibroblast Derived Induced Pluripotent Stem Cells" OR "Fibroblast-Derived IPS Cells" OR "Cell, Fibroblast-Derived IPS" OR "Cells, Fibroblast-Derived IPS" OR "Fibroblast-Derived IPS Cell" OR "Fibroblast Derived IPS Cells" OR "IPS Cell, Fibroblast-Derived" OR "IPS Cells, Fibroblast-Derived" OR "Human Induced Pluripotent Stem Cells" OR "Human Induced Pluripotent Stem Cell" OR "hiPSC"） |
| Ducement types | Article and Early access |
| Inclusion Criteria | Studies utilizing hiPSCs to model AD pathology |
| Exclusion Criteria | Non-English papers, Redundant publications, Titles/abstracts were focused on non-AD neurodegenerative diseases (e.g., Parkinson’s disease), Lacked explicit use of hiPSCs , non-research articles (e.g., editorials, conference abstracts) |
| Data extraction | Export with full records and cited references in plain text format |
| Sample size | 531 |

**S2 Table. Highly productive author in the hiPSCs model for AD research field.**

| Rank | Author | Documents | Citation | Average Citation |
| --- | --- | --- | --- | --- |
| 1 | Young, jessica e. | 13 | 535 | 41 |
| 2 | Koistinaho, jari | 12 | 358 | 30 |
| 3 | Zhao, jian | 11 | 378 | 34 |
| 4 | Tsai, li-huei | 11 | 1672 | 152 |
| 5 | Okano, hideyuki | 11 | 587 | 53 |
| 6 | Wang, ying | 10 | 14 | 1 |
| 7 | Young-pearse, tracy l. | 10 | 287 | 29 |

**S3 Table. Top 11 journals in the hiPSCs model for AD research field.**

| Rank | Source | Documents | Citations | Average Citation |
| --- | --- | --- | --- | --- |
| 1 | Stem cell research | 67 | 420 | 6 |
| 2 | Stem cell reports | 18 | 1014 | 56 |
| 3 | Scientific reports | 18 | 628 | 35 |
| 4 | Journal of Alzheimers disease | 15 | 283 | 19 |
| 5 | Cell reports | 14 | 579 | 41 |
| 6 | International journal of molecular sciences | 13 | 193 | 15 |
| 7 | Molecular psychiatry | 12 | 534 | 45 |
| 8 | Cells | 12 | 118 | 10 |
| 9 | Nature communications | 12 | 554 | 46 |
| 10 | Plos one | 10 | 942 | 94 |
| 11 | Molecular neurodegeneration | 10 | 386 | 39 |

**S4 Table. Journals partition of the hiPSCs model for AD research field.**

| Zone | Publication/Journal | Number of journals | Number of publications |
| --- | --- | --- | --- |
| 1 | ≥12 | 9 | 181 |
| 2 | 4-11 | 30 | 174 |
| 3 | 3-1 | 132 | 176 |

**S5 Table. Top 10 countries in the hiPSCs model for AD research field.**

| Rank | Country | Documents | Citations | Average Citation |
| --- | --- | --- | --- | --- |
| 1 | USA | 273 | 14015 | 51 |
| 2 | China | 80 | 1161 | 15 |
| 3 | Japan | 57 | 2207 | 39 |
| 4 | Germany | 57 | 1800 | 32 |
| 5 | England | 56 | 2557 | 46 |
| 6 | South Korea | 32 | 1191 | 37 |
| 7 | Sweden | 28 | 1134 | 41 |
| 8 | Austrilia | 24 | 687 | 29 |
| 9 | Italy | 19 | 689 | 36 |
| 10 | Denmark | 19 | 1296 | 68 |

**S6 Table. High-frequency keyword list in the reaearch field of hiPSCs model research for AD.**

| Rank | Keyword | Occurrence frequency | Total link strength |
| --- | --- | --- | --- |
| 1 | AD | 154 | 757 |
| 2 | Alzheimers - disease | 114 | 533 |
| 3 | Expression | 82 | 397 |
| 4 | pluripotent stem - cells | 70 | 342 |
| 5 | Brain | 60 | 300 |
| 6 | A - beta | 58 | 281 |
| 7 | Tau | 57 | 290 |
| 8 | Protein | 55 | 279 |
| 9 | Amyloid - beta | 55 | 275 |
| 10 | Neurons | 55 | 274 |
| 11 | Induced pluripotent stem cells | 54 | 279 |
